# Effects of kivia powder on Gut health in patients with occasional constipation: a randomized, double-blind, placebo-controlled study

**DOI:** 10.1186/1475-2891-12-78

**Published:** 2013-06-08

**Authors:** Jay K Udani, David W Bloom

**Affiliations:** 1 Medicus Research LLC, Agoura Hills, CA 91301, USA; 2 Medical Director, Northridge Hospital Integrative Medicine Program, Northridge, CA 91325, USA

**Keywords:** Constipation, Kiwi Fruit, Actinidin, Zyactinase™

## Abstract

**Objective:**

To evaluate the efficacy of Kivia powder on supporting overall gut health through the relief of the discomfort of occasional constipation.

**Design:**

Randomized, double-blind, placebo-controlled, parallel-group trial.

**Interventions:**

The investigational product for this study was Kivia powder (Vital Food Processors Ltd., Auckland, New Zealand), containing the active ingredient Zyactinase™, 5.5 g taken daily for four weeks.

**Results:**

One hundred thirty-eight subjects reporting occasional constipation were screened and 87 were randomized to placebo (n = 44) and product (n = 43). Bowel movement frequency, as measured by both average daily spontaneous bowel movements (SBM) and complete spontaneous bowel movements (CSBM), were the same in both groups at baseline. There were significant increases in spontaneous bowel movements at week 1 (p = 0.001), week 2 (p = 0.001), week 3 (p = 0.000), and week 4 (p = 0.000) compared to baseline. SBM demonstrated significant differences between the treatment group and the placebo group at week 3 (p = 0.000), and week 4 (p = 0.020). The treatment group demonstrated a significantly higher rate of SBM at week 3 (p = 000) and from baseline to week 4 (p = 0.019). Significant increases in complete spontaneous bowel movements were observed at week 1 (p = 0.000), week 2 (p = 0.000), week 3 (p = 0.000), and week 4 (p = 0.000) compared to baseline. Moreover, CSBM was significantly higher for the treatment group compared to placebo at week 2 (p = 0.001). The change in average daily CSBM from baseline to week 2 was significantly higher in the treatment group than in the placebo group (p = 0.004).

Abdominal discomfort or pain demonstrated significant differences between groups at week 1 (p = 0.044) and week 3 (p = 0.026). Flatulence was significantly lower for active group compared to placebo at week 2 (p = 0.047) and week 3 (p = 0.023). The number of bowel movements associated with urgency was significantly lower in the treatment group compared to the placebo group at week 3 (p = 0.048). In addition, it was decreased from baseline to week 1 (p = 0.040) and from baseline to week 3 (p = 0.024) in the treatment group, while the placebo group did not report any reductions in bowel urgency. Bowel movements in the treatment arm were significantly smoother and softer by week 2 (p = 0.020) and week 3 (p = 0.041).

**Conclusions:**

Treatment with Kivia powder, an extract of kiwifruit containing Zyactinase™, for four weeks was well tolerated and more effective than placebo in gently enhancing bowel movement frequency and reducing abdominal pain and flatulence in subjects with occasional constipation.

**Trial registration:**

ISRCTN: ISRCTN49036618

## Introduction

According to the American Gastroenterological Association, among healthy people, the frequency of bowel movements may vary from three movements per week to three daily. Constipation may be suspected if there is difficulty or pain when passing a hardened stool or if greater than three days pass between bowel movements. Occasional constipation is common and occurs in most people at some point. In some typically healthy individuals, evacuating the bowels is difficult at times, even in the absence of any physical or physiological symptoms. Neurological, psychological, or psychosomatic problems may be the cause in some individuals. Contributing factors to constipation include gender (being female), age (being older), diet and lifestyle choices, use of certain medications, and bowel habits.

In North America, constipation prevalence has been quoted at 1.9–27.2% [1,2], with the majority of the institutionalized elderly reporting laxative use on a daily basis [3] and women reporting constipation more frequently than men. In the United States, constipation is one of the top five outpatient gastrointestinal diagnoses [4], costing approximately $7,500 (US dollars) for diagnosis and treatment provision [5].

For the prevention and treatment of occasional constipation, the American Gastroenterological Association recommends eating a well-balanced diet, including bran, whole-wheat grains, and fresh fruit and vegetables, as well as drinking plenty of fluids and exercising regularly. It also recommends setting time aside for undisturbed visits to the toilet and not ignoring the urge to have a bowel movement. If these suggestions are not adequate, laxatives may be recommended by the physician. Over-the-counter laxatives for treatment of occasional constipation include bulk-forming laxatives, stimulants, stool softeners, lubricants, and osmotics.

Several herbal or natural products have been used traditionally or are of interest for constipation, including aloe, psyllium, probiotics, and rhubarb [6-8]. *Actinidia*, a genus of plants cultivated mainly for its fruit (kiwifruit), grows in various countries. Major producers of kiwifruit include Italy, New Zealand, Chile, and France. The fruit of the kiwi is of interest for gastrointestinal health, based on anecdotal data surrounding its cysteine protease constituent, actinidin. Preliminary published [9] and unpublished data suggest that use of kiwifruit decreased constipation in elderly patients. Rush et. al. reported the use of kiwifruit as laxative, including the increased frequency and ease of defecation, stool bulk, and softness in elderly patients [10]. Chan et. al. also investigated the benefits of kiwifruit in treating constipation in Chinese patients. The dietary fiber present in large amount in kiwifruit contributes to its laxative properties [11]. It has high water-containing capacity suggesting possible effect in fecal bulking and enhancement of laxation [12]. Several other clinical studies have explored the ability of zyactinase in alleviating constipation [13].

Kivia powder containing Zyactinase™ is a freeze-dried powder from kiwifruit (*Actinidia deliciosa* var. *deliciosa* (C.F. Liang and A.R. Ferguson), containing the enzyme actinidin, plant polyphenols, dietary fiber, carbohydrates, and oligosaccharides. Actinidin, an enzyme which can digest proteins, has been shown *in vitro* to dislodge meat boluses [14] and enhance gastric protein digestion [15,16]. In addition to the digestive enhancing action of actinidin, galactoglucomannan, a type of oligosaccharide, has also been isolated from kiwifruit [17]. It has been hypothesized that the oligosaccharides found in kiwifruit and Zyactinase™ may act as prebiotics in the human gut stimulating the growth of beneficial bacteria. Actinidin also aids in laxation by stimulating the receptors of the colon thus increasing colon motility [18].

Although the gastrointestinal effects of kiwifruit have been investigated in laboratory studies, there is a lack of human research in the area of constipation. This randomized, double-blind, placebo-controlled study was conducted to provide valuable data to support or refute the proposed effects of Kivia powder from kiwifruit on the improvement of gastrointestinal function in subjects with occasional constipation.

## Methods

### Investigational product

The investigational product for this study was Kivia powder (Vital Foods Processors Ltd, Auckland, New Zealand), containing the active ingredient Zyactinase™, at a dose of 5.5g per sachet. Kivia powder is an extract of kiwifruit (*Actinidia deliciosa* var. *deliciosa* (C.F. Liang and A.R. Ferguson)) prepared by a proprietary freeze-drying technique without solvents or extraction. The placebo was a combination of inactive components, including lemon powder, vital spirulina, citric acid, fructose, sucralose, and tropical flavor. Both products were provided by the sponsor, and both were GMP certified (batch numbers BN9460 and BN9465, for the product and placebo, respectively). The sachets were produced as identical packages. The daily dose of both groups was one sachet dissolved in cold water with breakfast. The sponsor also provided a rescue medication (bisacodyl rectal suppository, 10mg) for use during the study. Other over-the-counter products for constipation were not allowed during the course of the study. Also excluded were oral aloe vera and garlic supplements. Birth control hormonal agents and acetaminophen were allowed during the study.

### Subjects

Subjects included in the study were healthy adults, between 18 and 65 years of age and with a body mass index (BMI) between 20 and 35 kg/m^2^. The subjects had symptoms consistent with occasional constipation; these included at least two of the following occurring during the two-week run-in period: three or fewer defecations per week, straining during at least 25% of defecations, lumpy or hard stools in at least 25% of defecations, a sensation of incomplete evacuation for at least 25% of defecations, a sensation of anorectal obstruction or blockage for at least 25% of defecations, and manual maneuvers to facilitate at least 25% of defecations (e.g., digital evacuation, support of the pelvic floor). It is important to note that this was a study of dietary supplement and not a drug, hence any subject with a disease state related to the therapeutic area of the study product (including Irritable Bowel Syndrome (IBS)) was excluded from the study. Subjects with any significant gastrointestinal condition that would potentially interfere with the evaluation of the study product, including but not limited to, inflammatory bowel disease (ulcerative colitis or Crohn’s) were also excluded. Additional exclusion criteria included a history of perforation of the stomach or intestines, gastroparesis, or clinically important lactose intolerance, known allergy or sensitivity to kiwi fruit, or a recent (within two weeks of visit 1, week -1) episode of acute gastrointestinal illness such as nausea, vomiting, or diarrhea were excluded. Subjects were also required to be willing to maintain his or her habitual food and beverage intake and physical activity patterns throughout the study period. The subjects were judged by the investigator to be in general good health on the basis of their medical histories. Informed consents were signed and returned prior to dispensing any study product. In addition, any concomitant medications and/or dietary supplements, which affected the GI tract, were prohibited during the study. Subjects were recruited from the community, including through online recruiting (Craigslist), advertising, and available databases. Subjects were phone-screened prior to scheduling a screening visit.

### Study design

**Figure 1:**
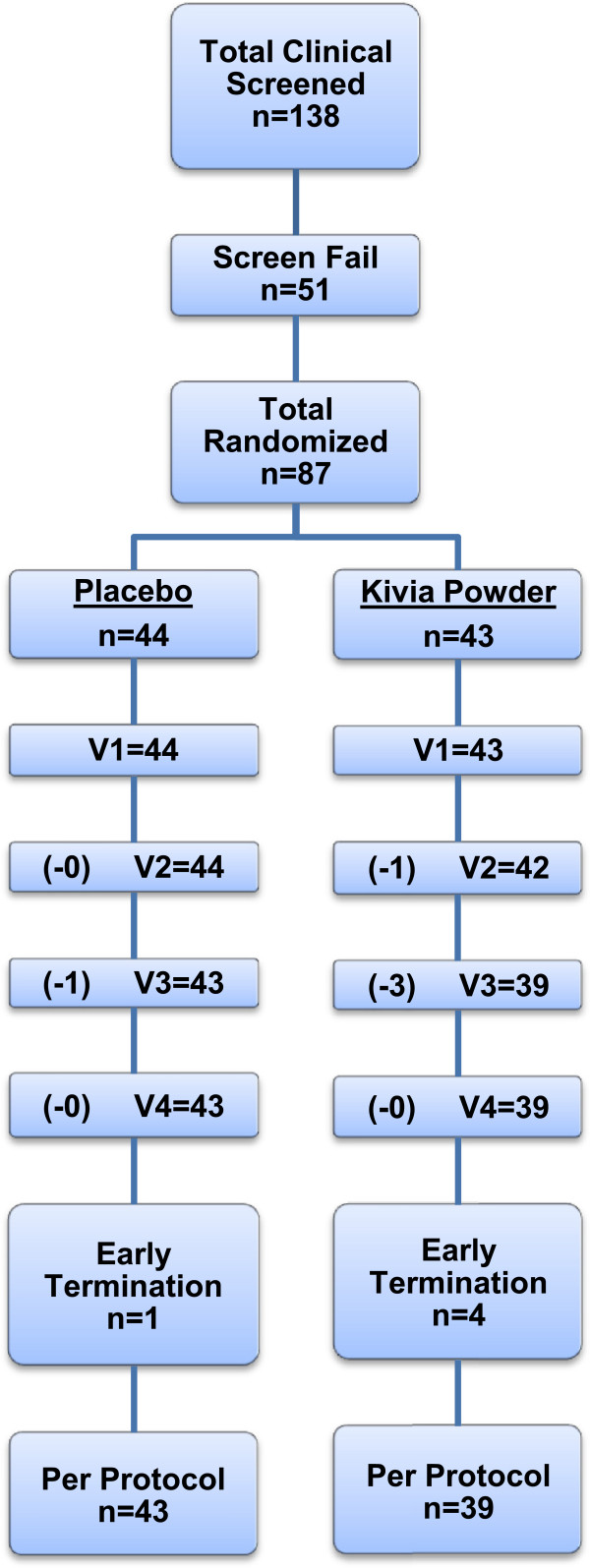
Study procedure flowchart.

This was a randomized, double-blind, placebo-controlled, parallel-design study, with 87 men and women taking the study product daily for four weeks. An interim analysis on the primary endpoint was performed after completion by the first 50 subjects. It was determined that additional subjects would be necessary to reach statistical significance. Simple randomization was prepared using a computer program based on the atmospheric noise method, and sequential assignment was used to determine group allocation. Group allocation was placed in individual numbered envelopes, to maintain blinding of all individuals. The study was double-blind, using identical sachets. Subjects, clinical staff, data management staff, and statistical analysis staff were unaware of the study group. The study was conducted at the Staywell Research clinical research site located in Northridge, CA, and Medicus Research was the contract research organization (CRO) for this study. Subjects were recruited from the community and assigned to treatment by the research site. Institutional review board (IRB) approval was received (Copernicus Group IRB, Cary, NC) prior to the initiation of any study-related activities (approved August 2010, first patient recruited September 2010, last patient recruited October 2011). The study flowchart is provided in Figure 1. Five subjects did not complete the study, because they were lost to follow-up (the patients stopped coming in and no reason was provided). The trial concluded following randomization and completion of the required number of subjects.

At screening (visit 1, day −14), subjects underwent the informed consent process and were screened for the presence of all of the inclusion criteria and the absence of all of the exclusion criteria. This screening process included a detailed medical history, vital signs, anthropometric measures, demographic data, and urine collection for pregnancy (if applicable). If subjects were deemed eligible, they were asked to stop the use of any medications for constipation and to begin keeping a daily diary of their bowel habits.

At baseline (visit 2, day 0), eligibility was reconfirmed with regard to medical history and concomitant medication history. If the subjects met all of the inclusion criteria and none of the exclusion criteria, they were randomized based on an envelope containing the appropriate group placement (based on a previous randomization scheme). At this visit, the subjects were instructed verbally on the following: the use and storage of the study product; the number, dates, and times of visits; any study procedures; daytime and emergency contact information; completion of the study diary; and returning unused product.

At week 2 (visit 3, day 14), subjects returned to the study site. Medical histories and medication histories were reviewed and updated. At this time, completed diaries and unused product were returned and obligations were discussed. Subjects were reminded verbally on the use and storage of the study product, as well as the number, dates, and times of visits, any study procedures, daytime and emergency contact information, completion of the study diary, and returning unused product.

At week 4 (visit 4, day 30), subjects returned to the study site. Medical histories and medication histories were reviewed and updated. At this time, completed diaries and unused product were returned. This visit included vital signs and anthropometric measures.

Subjects were called twice by the study staff in order to improve compliance and completion of the study diary, to remind subjects of their obligations and upcoming visits, and to discuss any medical issues.

### Endpoints

This study was designed to determine the efficacy of Kivia powder on the improvement of gut health parameters in subjects with occasional constipation.

#### Primary endpoint

The primary endpoint of this study was bowel movement frequency. Bowel movement frequency is a parameter used to assess how often an individual have his/her bowel movement and is reported as a daily average by week. The numbers of spontaneous bowel movements and complete spontaneous bowel movements were investigated. Spontaneous bowel movement (SBM) is defined as a stool not induced by rescue medication while complete spontaneous bowel movement (CSBM) is SBM associated with a sensation of complete evacuation. In addition, a complete spontaneous bowel is a bowel movement during which the subject answered “yes” to the question “Have you completely emptied your bowel?”.

#### Secondary endpoints

The secondary objective was to determine the efficacy of Kivia powder compared to placebo on gut health, based on diary information. Endpoints included stool form (Bristol Stool Scale), bowel urgency (yes/no), abdominal bloating (0 = none, 1 = mild, 2 = moderate, 3 = intense, 4 = severe), abdominal discomfort or pain (0 = none, 1 = mild, 2 = moderate, 3 = intense, 4 = severe), satisfaction with bowel habits (0 = a great deal satisfied, 1 = a good deal satisfied, 2 = moderately satisfied, 3 = hardly satisfied, 4 = not satisfied at all), flatulence (0 = none, 1 = mild, 2 = moderate, 3 = intense, 4 = severe), and burping (0 = none, 1 = mild, 2 = moderate, 3 = intense, 4 = severe). The Bristol Stool Scale is used to classify the form of feces into categories (type 1 = separate hard lumps, like nuts, type 2 = sausage-shaped but lumpy, type 3 = like a sausage with cracks on surface, type 4 = like a sausage or snake, smooth and soft, type 5 = soft blobs with clear-cut edges and passed easily, type 6 = fluffy pieces with ragged edges, a mushy stool, type 7 = watery, no solid pieces, entirely liquid) [14].

#### Tertiary endpoints

The tertiary objective of the study was to determine the efficacy of Kivia powder compared to placebo on the use of rescue medication.

#### Quaternary endpoints

The quaternary objective of the study was to determine the safety of Kivia powder compared to placebo. This was based on an adverse event assessment.

### Statistics

A modified per-protocol analysis was performed, including all subjects who had at least one poststudy product exposure visit. Primary endpoints were analyzed where possible, both within groups and between groups. A paired sample t-test was used to assess changes over time for each group. All data elements were screened for reasonableness, and all missing, suspicious, or impossible values were referred back to the monitoring team for query generation and resolution. All numerical variables were tested for normality, and data found to be substantially non-normally distributed were analyzed by appropriate nonparametric methods. Numerical variables were summarized as the number of subjects, mean, standard deviation, and significance. Categorical variables were summarized as counts and percentages. Data were derived from diary entries, clinical assessments, questionnaires, and other relevant assessments at postbaseline measurement points.

An interim analysis on the primary endpoint was performed after the completion of the first 50 subjects. It was determined that additional subjects would be necessary to reach statistical significance. All study and data personnel remained blinded until the conclusion of the analysis, at which point only the statistician was unblinded. The efficacy of test products was evaluated with respect to stool form, bowel urgency, abdominal bloating, abdominal discomfort or pain, satisfaction with bowel habits, flatulence, and burping. Summary statistics (n, mean, standard deviation (SD) and standard error (SE) means) were presented for each of these numeric secondary outcome variables by product. Paired t-tests were employed to check for any difference between baseline and each assessment time point in these parameters. Independent t-test was employed to assess the efficacy of the products.

Excel 2003 (Microsoft Corp., Redmond, WA) was used for data entry, validation, restructuring, calculating changes in variables over time, reorganizing and reformatting results, and preparing graphs. Statistical analyses (descriptive statistics and Student’s t-tests) were performed using SPSS Base System ver. 19 (IBM SPSS Inc., Chicago, IL). Data were analyzed between groups using independent sample t-tests to determine the change from baseline. Statistical analyses were performed using SPSS Base System ver. 19. Significance was indicated at p < 0.05.

## Results

One hundred thirty-eight subjects were screened to take part in this study. Of these, 87 were randomized to placebo (n = 44) and product (n = 43). There were four withdrawals in the active group and one in the placebo group. These five patients were lost to follow-up (the patients failed to show for visits). Therefore, the results provided are for 82 subjects. The demographics and baseline characteristics are shown in Table 1.

**Table 1:** Demographic and baseline characteristics

**Baseline characteristics**	**Placebo**	**Active**	**Significance**
N	44	43	
Male	18	14	
Female	26	29	
Age (Mean)	41 ± 14	38 ± 14	0.236
Age (Range)	20-64	19-62	
Weight (Mean)	164.79 ± 29.05	158.29 ± 33.71	0.341
Weight (Range)	114.8-222.0	110.0-235.0	
BMI (Mean)	27 ± 4.11	25.8 ± 4	0.165
BMI (Range)	19.2-34.8	20.1-35.0	
**Marital status**			
Single	18	22	
Married	22	17	
Divorced	4	3	
Separated	0	1	
Widowed	0	0	
Domestic Partner	0	0	
**Ethnicity**			
White	9	14	
Asian	0	0	
African-American	2	5	
Hispanic	30	23	
American Indian/ Alaska Native	0	0	
Hawaiian/ Pacific Islander	0	0	
Other	3	1	

There were no significant differences in body temperature, diastolic blood pressure, pulse, or respiratory rate between groups or compared with baseline. Systolic blood pressure was statistically but not clinically significant different between groups at visit 3 (115.10 mmHg in the active group vs. 122.34 mmHg in the placebo group (p = 0.02)). There were no significant differences in weight between groups at any time point (p > 0.05). BMI was significantly lower in the treatment group at week 4 compared to the placebo group (25.36 kg/m^2^ vs. 27.23 kg/m^2^, p = 0.037), with no significant changes compared to baseline in either group (p > 0.05).

**Figure 2:**
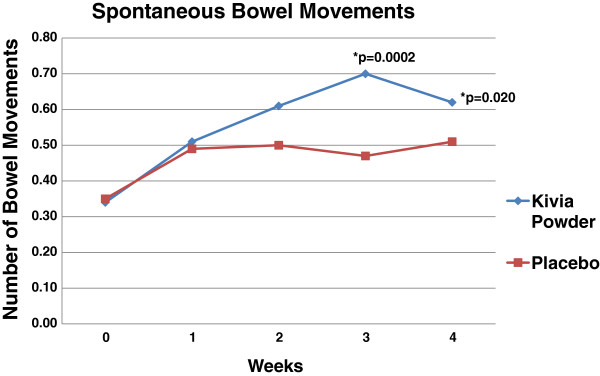
**Comparison of Kivia powder and placebo in spontaneous bowel movement.** Spontaneous bowel movement was significantly higher for Kivia Powder group compared to placebo group at week 3 and week 4.

**Figure 3:**
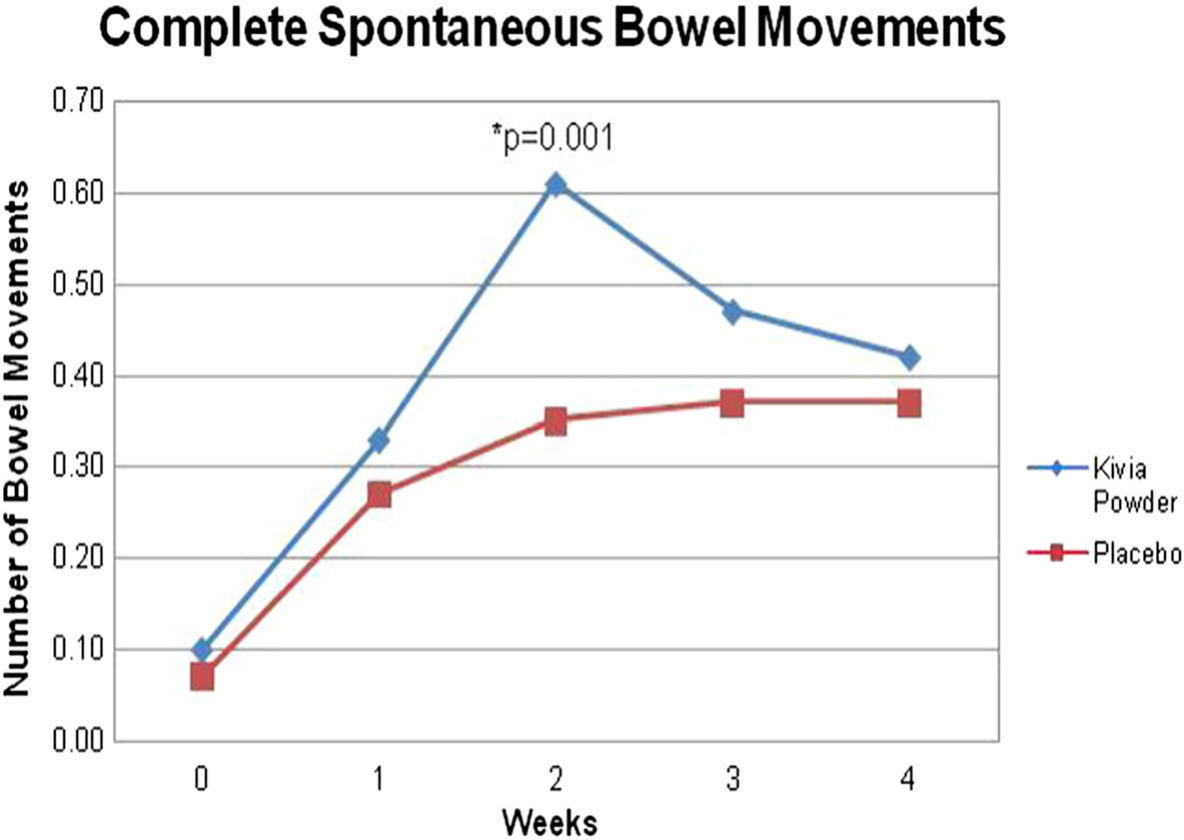
**Comparison of Kivia powder and placebo in complete spontaneous bowel movement.** Complete spontaneous bowel movement was significantly higher for Kivia Powder group compared to placebo group at week 2.

The primary endpoint of this study was bowel movement frequency. The numbers of spontaneous bowel movements and complete spontaneous bowel movements were investigated. The subjects in the active group demonstrated significant increases in number of spontaneous bowel movements (SBM) at week 1 (p = 0.001), week 2 (p = 0.001), week 3 (p = 0.000), and week 4 (p = 0.000) compared to baseline. In addition, the SBM endpoint demonstrated nearly significant and significant differences between the active and the placebo group at week 2 (p = 0.077), week 3 (p = 0.000), and week 4 (p = 0.020) (Table 2, Figure 2). The changes in SBM from baseline to week 3 (p = 0.000) and from baseline to week 4 (p = 0.019) were significantly higher in the treatment group compared to the placebo group. Significant increases in number of complete spontaneous bowel movements (CSBM) were observed at week 1 (p = 0.000), week 2 (p = 0.000), week 3 (p = 0.000), and week 4 (p = 0.000) compared to baseline. Moreover, CSBM was significantly higher for active group compared to placebo at week 2 (p = 0.001) (Table 3, Figure 3). The change in daily average of CSBM from baseline to week 2 (p = 0.004) was significantly higher in the treatment group compared to the placebo group.

**Table 2:** Effect of treatment on daily bowel movement frequency (Spontaneous Bowel Movements)

	**Baseline (mean ± SE)**	**Week 1 (mean ± SE)**	**Week 2 (mean ± SE)**	**Week 3 (mean ± SE)**	**Week 4 (mean ± SE)**
**Placebo**	0.35 ± 0.012	0.49 ± 0.035	0.50 ± 0.039	0.47 ± 0.026	0.51 ± 0.029
**Kivia powder**	0.34 ± 0.012	0.51 ± 0.044	0.61 ± 0.043	0.70 ± 0.054^a^	0.62 ± 0.038^b^

^a^p ≤ 0.01 vs. placebo at this time point.

^b^p ≤ 0.05 vs. placebo at this time point.

**Table 3:** Effect of treatment on daily bowel movement frequency (Complete Spontaneous Bowel Movements)

	**Baseline (mean ± SE)**	**Week 1 (mean ± SE)**	**Week 2 (mean ± SE)**	**Week 3 (mean ± SE)**	**Week 4 (mean ± SE)**
**Placebo**	0.07 ± 0.016	0.27 ± 0.046	0.35 ± 0.063	0.37 ± 0.059	0.37 ± 0.055
**Kivia powder**	0.10 ± 0.020	0.33 ± 0.044	0.61 ± 0.043^a^	0.47 ± 0.051	0.42 ± 0.057

^a^p≤0.01 vs. placebo at this time point.

**Figure 4:**
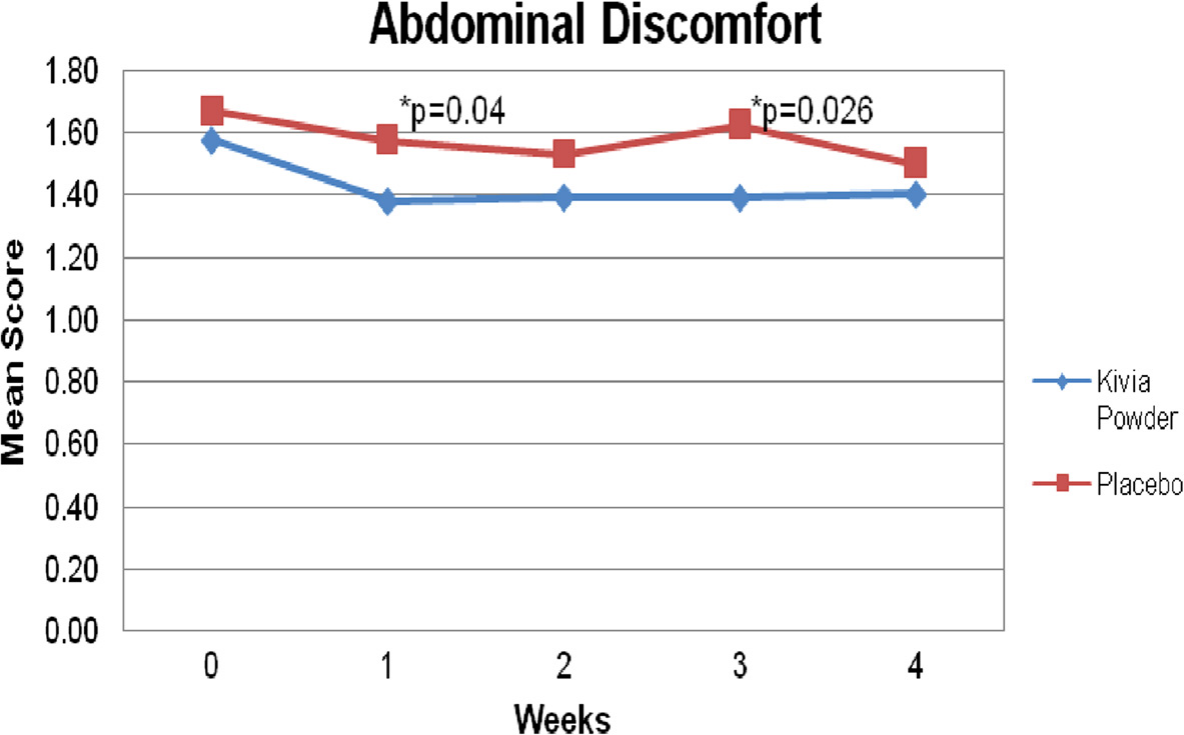
**Comparison of Kivia powder and placebo in abdominal discomfort.** Abdominal Discomfort was significantly lower for Kivia Powder group compared to placebo group at week 1 and week 3.

The secondary objective of this study was to determine the efficacy of Kivia powder on gut health compared to placebo. There were nearly significant and significant decreases in abdominal bloating at week 1 (p = 0.095), week 2 (p = 0.003), week 3 (p = 0.002), week 4 (p = 0.004) compared to baseline. Significant and nearly significant reductions in abdominal discomfort or pain were observed at week 2 (p = 0.059) and week 4 (p = 0.018) compared to baseline. Moreover, abdominal discomfort or pain was lower for the treatment group compared to placebo at week 1 (p = 0.044) and week 3 (p = 0.026) (Figure 4). There were significant increases in satisfaction with bowel habits at week 1 (p = 0.001), week 2 (p = 0.000), week 3 (p = 0.000), and week 4 (p = 0.000) compared to baseline. There was no significant difference between groups in terms of abdominal bloating and satisfaction with bowel habits.

**Figure 5:**
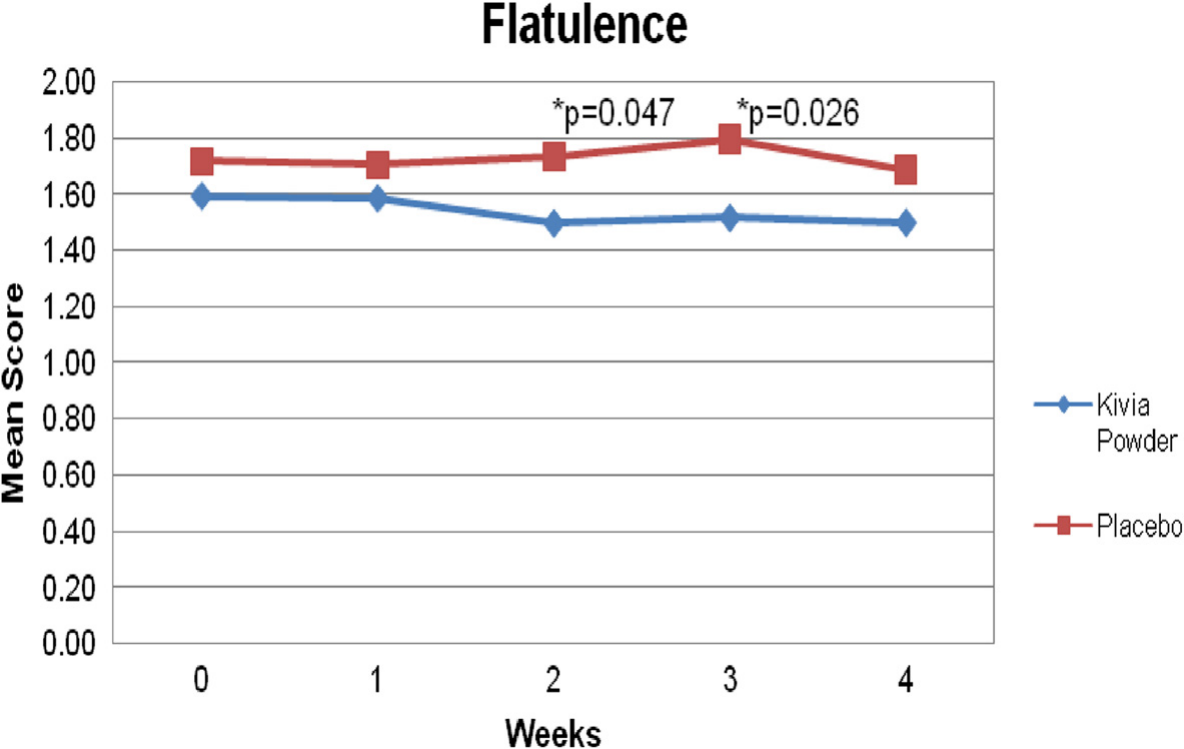
**Comparison of Kivia powder and placebo in flatulence.** Flatulence was significantly lower for Kivia Powder group compared to placebo group at week 2 and week 3.

Flatulence was significantly lower for active group compared to placebo at week 2 (p = 0.047) and week 3 (p = 0.023) (Figure 5). Burping demonstrated nearly significant difference between the active and the placebo group at week 4 (p = 0.079).

Bowel movement associated with urgency was reduced in the treatment group compared to the placebo group from weeks 1 to 4. The percent reduction in bowel urgency in the active group compared to the placebo group at weeks 1, 2, 3, and 4 were −26%, -25%, -32%, and −26%, respectively. The number of bowel movements associated with urgency was significantly lower in the treatment group compared to the placebo group at week 3 (p = 0.048). Moreover, the number of bowel movements associated with urgency decreased from baseline to week 1 (p = 0.040) and to week 3 (p = 0.024) in the treatment group, while the placebo group did not report any reductions in bowel urgency.

In terms of stool forms, there was a reduction in stool forms 1 and 2 and an increase in stool forms 3, 4, and 5 in both groups. Between-group comparisons demonstrated that by week 2, there were significantly more type 4 bowel movements (sausage-shaped and smooth) in the Kivia powder group compared to the placebo group (p = 0.020), and by week 3, there were significantly more type 5 bowel movements (soft blobs with clear-cut edges) in the treatment group compared to the placebo group (p = 0.041).

There were no serious adverse events reported in this study. There were a total of eight adverse events. Seven of the eight adverse events occurred in the treatment group. Five of the seven adverse events in the treatment group were considered as being possibly related to the treatment, and these included flatulence (MedDRA diagnosis code 10016766; n = 3) and bloating (MedDRA diagnosis code 10005265; n = 2). These adverse events resolved spontaneously without treatment.

## Discussion

In this study, an extract of kiwifruit (Kivia powder) containing Zyactinase™ was investigated for its ability to improve bowel function and satisfaction in subjects with occasional constipation. Bowel function was determined using various endpoints, including the number of bowel movements, and bowel health was based on endpoints such as satisfaction, abdominal bloating and discomfort, burping, and flatulence. Improvements were noted in the number of bowel movements, with increased bowel movements in the group using the studied extract. There were also improvements observed in bowel health and stool formation. This suggests that Kivia powder improved bowel habits in this group of subjects.

Chronic constipation is often dealt with by a physician with recommendations for over-the-counter medications such as bulk-forming laxatives, stimulants, stool softeners, lubricants, or osmotics. Although individuals suffering from occasional constipation also have access to and use these agents, with or without knowledge of their physicians, many individuals might prefer the option of an effective natural agent for occasional use. Given the high costs associated with the diagnosis and treatment of constipation [5] in conjunction with considerable prevalence rates noted in the literature [4], it is clear that these products would be of interest to consumers. High-fiber food products and supplements containing psyllium or flax, as well as aloe and probiotics, are sold and used for this purpose and have been investigated in clinical trials [6-8]. Kivia powder containing Zyactinase™ is a freeze-dried powder from kiwifruit (*Actinidia deliciosa* var. *deliciosa* (C.F. Liang and A.R. Ferguson), containing the enzyme actinidin, plant polyphenols, dietary fiber, carbohydrates, and oligosaccharides. Although the potential mechanism of action for its anticonstipative effects are not yet completely clear, kiwifruit extract is rich in enzymes able to aid in digestion, such as actinidin, as well as oligosaccharides, which may act as prebiotics, enhancing the growth of beneficial bacteria in the gastrointestinal tract. Additionally, *in vitro*, Zyactinase™ significantly increased the growth of the probiotic bacteria *Lactobacillus reuteri*, *Lactobacillus acidophilus*, *Pediococcus acidilactici*, and *Lactobacillus planetarium*, further supporting its prebiotic role in the gut [18]. In this study Kivia aided gentle laxation without urgency whilst promoting normal stool formation, making the product ideally suited as an over the counter medication for occasional constipation.

Although strong published human and animal data are lacking at this time, unpublished data suggest that use of the fruit decreased constipation in elderly patients in New Zealand. Also, in an earlier clinical study, Kivia was found to promote bowel movement, improve discomfort in the abdomen, and improve quality of life in elderly patients in Japan [9]. Like the study by Uebaba et al., our study also suggests improvement in the number of bowel movements. Further research is required in order to compare our results with those of others.

The main endpoints of this study included the number of spontaneous and complete spontaneous bowel movements. Both spontaneous and complete spontaneous bowel movement frequency increased between groups and compared to baseline and these improvements are likely due to the multiple effects of the extract on bowel health as described above. Secondary endpoints of this study included abdominal bloating, abdominal discomfort or pain, satisfaction with bowel habits, flatulence, burping, stool form and bowel urgency. Abdominal bloating, abdominal discomfort or pain, and satisfaction with bowel habits demonstrated significant decreases from baseline. In addition, abdominal pain or discomfort and flatulence were significantly lower in the treatment group than in the placebo group. Stool form was also improved as proven by the decrease in stool form 1 and 2, which are characteristic stool of people with constipation, and an increase in stool form 3 and 4, the optimal stool form, in the treatment group. These results suggest that Kivia may be of assistance in other gut conditions related to these secondary gut health parameters such as irritable bowel syndrome and chronic constipation, but that will require further study.

Regarding within group change over time, satisfaction and abdominal bloating did not improve significantly in either group. However, abdominal bloating steadily decreased and satisfaction steadily increased in both groups nonsignificantly with time. This suggests that taking part in the study itself may have allowed for some placebo-related benefits and that additional benefits of the product were not obvious. Also, expectations play a role in satisfaction, and the expectations of the study product may have been high. It is interesting to note the strong placebo effect so often reported with gut health studies and that not only did bowel movements increase but also that the stool features also improved in the placebo group albeit not as much as the active group

Bowel movement frequency is an objective endpoint and easily quantified compared to subjective feelings of associated symptoms. It is a common misconception that normal bowel habits involve daily excretion [5] and this misconception may have contributed to a lack of improvement in satisfaction despite increased bowel movements. Furthermore, since subjects were not screened and selected based on secondary gut health parameters, variability within our sample along these endpoints would have contributed to decreased room for improvement thus any improvement is noteworthy and lends credence to further exploration.

Another limitation of this study was that the subjects were not specifically chosen based on gut health parameters such as bloating, pain, and flatulence. Given that these symptoms are also associated with irritable bowel syndrome, future studies should screen for these symptoms and only subjects with certain levels of these symptoms should be included. Thus, further research is required to evaluate the impact of the Kivia powder product on a population of subjects who would be specifically selected for their baseline level of other gut health parameters (bloating, pain, flatulence, burping, etc.) to determine the true impact of the product on these symptoms.

In conclusion, this proprietary extract of kiwifruit, containing Zyactinase™, significantly increased the number of spontaneous and complete spontaneous bowel movements after four weeks when compared to placebo. Gut health parameters associated to constipation also improved from baseline and within groups. In addition, the product was well tolerated at the daily-administered dose of one sachet (containing 5,500 mg of Zyactinase™). Also, the decreased use of rescue medication in the treatment group, already lower than in the placebo group at baseline, suggests that the product was helpful in this group of individuals. The results of this study suggest that this product may be of interest to generally healthy adults with occasional constipation and associated abdominal discomfort.

## Abbreviations

AUC: Area under the curve; BMI: Body mass index; CRO: Contract research organization; CSBM: Complete spontaneous bowel movements; SBM: Spontaneous bowel movements; IRB: Institutional review board.

## Competing interests

Medicus Research has ongoing research support grants from Vital Food Processors Ltd. Auckland New Zealand. Dr. Udani has provided consulting services to Vital Food Processors Ltd. The authors and Medicus Research do not endorse any brand or product.

## Authors’ contributions

JKU was the Principal Investigator. DWB contributed to writing the manuscript and assisted with data interpretation. Both authors read and approved the final manuscript.

## Authors’ information

JKU was the Principal Investigator, was responsible for the design of the study, data interpretation, and manuscript writing. DWB was a scientific coordinator working at Medicus Research at the time of the study and contributed to writing the manuscript and assisted with data interpretation.
